# Inhibition of *Burkholderia multivorans* Adhesion to Lung Epithelial Cells by Bivalent Lactosides

**DOI:** 10.3390/molecules170910065

**Published:** 2012-08-24

**Authors:** Ciara Wright, Rosaria Leyden, Paul V. Murphy, Máire Callaghan, Trinidad Velasco-Torrijos, Siobhán McClean

**Affiliations:** 1 Centre of Microbial Host Interactions, Institute of Technology Tallaght Dublin, Belgard Road, Tallaght, Dublin 24, Ireland; 2 Department of Chemistry, National University of Ireland, Galway, Ireland; 3 Department of Chemistry, National University of Ireland, Maynooth, Co., Kildare, Ireland

**Keywords:** bacterial colonization, bivalent lactoside, *Burkholderia cepacia* complex, cystic fibrosis, glycoconjugate

## Abstract

*Burkholderia cepacia* complex (Bcc) is an opportunistic pathogen in cystic fibrosis patients which is inherently resistant to antimicrobial agents. The mechanisms of attachment and pathogenesis of Bcc, a group of 17 species, are poorly understood. The most commonly identified Bcc species in newly colonised patients, *Burkholderia multivorans*, continues to be acquired from the environment. Development of therapies which can prevent or reduce the risk of colonization on exposure to Bcc in the environment would be a better alternative to antimicrobial agents. Previously, it has been shown that Bcc strains bound to many glycolipid receptors on lung epithelia. Using a real-time PCR method to quantify the levels of binding of *B. multivorans* to the lung epithelial cells, we have examined glycoconjugate derivatives for their potential to inhibit host cell attachment. Bivalent lactosides previously shown to inhibit galectin binding significantly reduced the attachment of *B. multivorans* to CF lung epithelial cells at micromolar concentrations. This was in contrast to monosaccharides and lactose, which were only effective in the millimolar range. Development of glycoconjugate therapies such as these, which inhibit attachment to lung epithelial cells, represent an alternative means of preventing infection with inherently antimicrobially resistant pathogens such as *B. multivorans*.

## 1. Introduction

Cystic fibrosis (CF) is a genetically inherited disease that is characterised by chronic lung infections which have a major impact on the mortality and quality of life of these patients. The majority of the opportunistic pathogens acquired by CF patients in later years are inherently antimicrobial resistant. These include *Pseudomonas aeruginosa* and members of the *Burkholderia cepacia* complex (Bcc). This latter pathogen is a group of 17 species, all of which are characterised by having multiple mechanisms of resistance to multiple antibiotics [1], and consequently this pathogen is rarely eradicated once a patient has been colonised. Although strict segregation measures have minimized patient-to-patient spread, Bcc, and increasingly *B. multivorans*, continues to be acquired from the environment [2]. Prevention of colonisation by topical application of biodegradable binding inhibition agents may represent a more effective approach than antibiotic therapy [3].

Previously, it has been shown that Bcc strains bind to many glycolipid receptors, including asialoGM1, asialoGM2 and globosides, on lung epithelia [4,5]. We have subsequently shown that intracellular invasion of *B. multivorans* into human lung cells relied on prior attachment to glycosphingolipids and not mucins [6]. However, the complete elucidation and identification of these receptors has yet to be determined. In the absence of clear identification of the actual receptors, screening a range of glycoconjugates for their potential to inhibit or compete with Bcc for binding sites, represents an alternative approach to the discovery of prophylactic treatments to prevent colonization. We have developed a rapid, reliable quantitative PCR technique for the identification of Bcc adhering to lung epithelial cells *in vitro* and have previously shown that millimolar concentrations of lactose could significantly decrease bacterial attachment to lung cells by over 50% [7].

Leyden *et al.*, described the preparation of bivalent lactosides based on terephthalamides and glycophane scaffolds and demonstrated that they inhibited galectins, most readily galectin-3 [8]. Both flexible and rigid lactosides were potent at inhibiting binding of galectins to asialofetuin, while the more flexible bivalent lactosides blocked plant toxin binding to cells more efficiently than those with a more rigid scaffold. In this Communication, we further examine whether three of these galectin inhibitors could inhibit attachment of *B. multivorans* to lung epithelial cells. 

## 2. Results and Discussion

**Figure 1:**
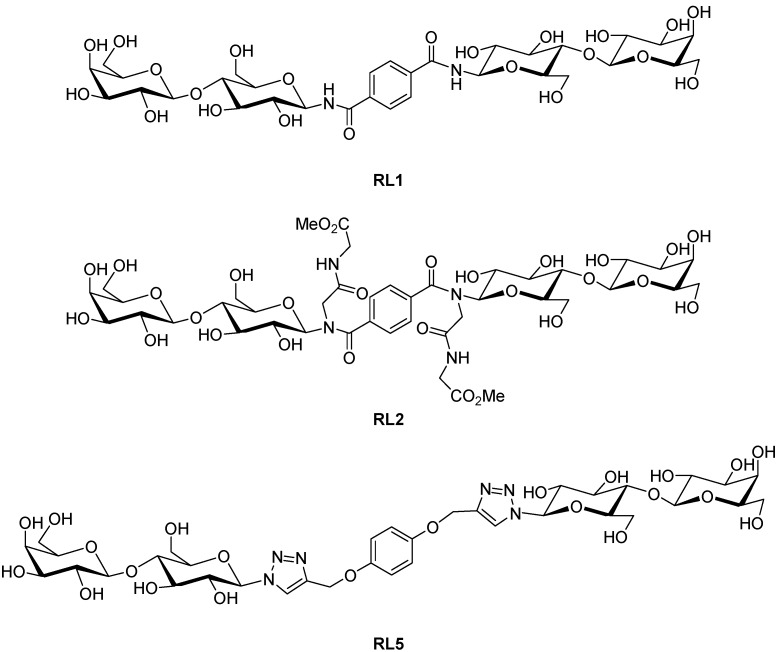
Chemical structure of bivalent lactosides used in this study. (**a**) RL1; (**b**) RL2 and (**c**) RL5.

The real-time PCR method has previously been developed and validated to quantitatively determine the attachment of *B. multivorans* to lung epithelial cells and its subsequent inhibition with simple sugars [7]. We chose two rigid and one flexible bivalent lactosides to investigate whether more complex glycoconjugates would be more effective inhibitors of bacterial attachment (Figure 1). RL1, the secondary amide was previously shown to have a constrained interlactose distance of 10.1 to 10.4 Å [8]. RL2, the tertiary amide has an interlactose distance which is constrained between 7.2 and 8.1 Å. In contrast, RL5 is very flexible with an interlactose distance which varies from 9 to 17 Å. Bacteria were applied to lung epithelial cells in the absence and presence of glycoconjugates, allowed to attach and unattached bacteria washed off. The amount of remaining bacterial DNA was determined by quantitatitve real time PCR and normalized with human DNA using the glyceraldehyde phosphate dehydrogenase (GAPDH) gene.

**Figure 2:**
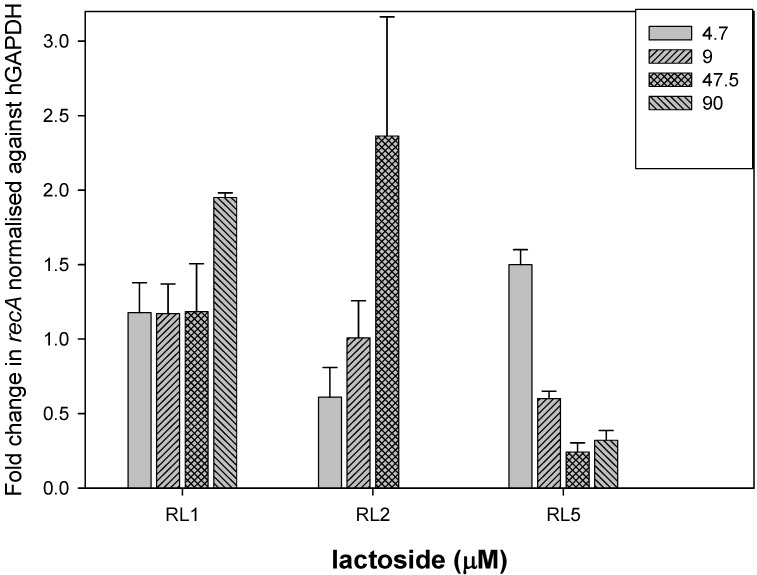
Inhibition of binding of *B. multivorans* to lung epithelial cells. Bacteria were pre-incubated with lactosides for 30 min prior to infection of lung epithelial cells. Bars represent the fold change in binding of bacteria to lung epithelial cells relative to uninhibited solvent control.

Preincubation of the bacteria with the flexible linker, RL5, strongly inhibited binding of *B. multivorans* to lung epithelial cells at concentrations from 9 to 90 μM (Figure 2). In contrast, the rigid lactoside, RL1, had little effect on binding of *B. multivorans* to lung cells at lower concentrations and indeed enhanced the amount of bacteria in contact with the cells by two-fold at the highest concentration tested. Although, the tertiary amide with the shortest interlactose distance, RL2, reduced attachment at low micromolar concentrations, it increased the levels of bacteria attached relative to uninhibited control by 2.5 fold at 47 μM. In previous studies, we showed that lactose reduced attachment of *B. multivorans* to lung cells by 50% at concentrations of 20 mM, but had little effect at 1 to 5 mM, while under the same conditions, galactose had no effect on bacterial attachment [7]. This study shows that the flexible bivalent lactoside is much more effective than lactose at inhibiting binding of *B. multivorans* to host cells.

The effect of the flexible lactoside at micromolar concentrations is promising in terms of future development of bacterial colonization inhibitors. Leyden *et al*. have previously shown that RL5 resulted in marked inhibition of plant toxin binding to human lymphoblastoid cells, being more effective than lactose, and that the less flexible lactosides were less potent than that of lactose [8]. This study is in agreement with this, and again shows that the flexible bivalent lactoside is a potent inhibitor of bacterial binding. The increase in bacteria detected at higher concentrations of RL1 and RL2 was unexpected. It is possible that the conformation of these two lactosides allows the lactose moieties at either end bind to both bacteria and host cell simultaneously therefore acting as a bridge between the cells and enhancing their attachment. It is not known what affinity the lactosides have for host cells and this will be assessed in future studies.

It has previously shown that another species in the Bcc, *B. cenocepacia*, expresses four soluble lectins with affinities for mannose or fucose, including a superlectin, BCL2-C, with dual specificities, fucose binding at the N-terminal domain with mannose and heptose affinity at the C-terminal domain [9]. However, the expression of lectin across other members of the Bcc, or in *B. multivorans*, specifically, has not been reported. Although *B. cenocepacia* is the most virulent of species within the Bcc, *B. multivorans* is currently being isolated more frequently from CF patients both in Europe and the US than *B. cenocepacia* [10]. A study by Baldwin *et al*. on *B. multivorans* isolates from industrial products and water environments showed that these were indistinguishable from clinical isolates, indicating that environmental sources may act as an important reservoir for infection of these species [11]. It is therefore important to be able to block the colonization of this species in CF patients.

Further studies are ongoing to evaluate several aspects of these glycoconjugates, including development of alternative carbohydrate moieties on the same scaffold, in addition to examining analogues of the flexible lactoside. In addition, the specificity of these glycoconjugates for inhibition of *Burkholderia* species needs to be assessed. This initial study shows promise for the future development of inhibitors of bacterial attachment in prevention of colonization by difficult to treat antimicrobial resistant pathogens.

## 3. Experimental

### 3.1. Preparation of Compounds

The bivalent lactosides were prepared as outlined previously [8]. They were dissolved in DMSO which was subsequently diluted with Luria Bertani (LB) broth prior to analysis.

### 3.2. Bacterial Strain and Epithelial Cells

*B. multivorans* strain LMG13010, a member of the International *Burkholderia cepacia* working group panel was obtained from the BCCM/LMG University of Ghent (Ghent, Belgium) and routinely cultured on LB broth at 37 °C. Overnight cultures were grown to an OD of 0.6 and diluted. The lung epithelial cell line 16HBE14o− was kindly donated by Dr. Dieter Gruenert (University of California, San Francisco, San Francisco, CA, USA) and maintained in fibronectin-vitrogen coated flasks containing minimal essential medium (MEM) plus 10% (v/v) FBS as described previously [12]. For adhesion assays, 16HBE14o− cells were seeded on coated 24 well plates at 2.5 × 10^5^ cells·mL^−1^ in supplemented MEM in the absence of antibiotics and incubated overnight at 37 °C in 5% CO_2_. The bacteria (~5 × 10^6^ CFU) were then applied to the cells in MEM only and the plate was centrifuged at 700 *g* for 5 min, to facilitate bacterial attachment. The plate was incubated at 37 °C in the presence of 5% CO_2_ for 30 min and then washed vigorously three times with PBS to remove unbound bacteria. 

### 3.3. Relative Quantitation of B. multivorans

The bacteria attached to the lung cells were detected by quantitative real time PCR method as described in Wright *et al*. [7]. DNA was extracted from the cells using a Wizard^®^ Genomic DNA purification kit (Promega, Southampton, England) as outlined in the manufacturer’s instruction, to purify the template prior to amplification. The GAPDH gene was used as a reference to normalise the level of bacterial DNA present with that of human DNA. Forward and reverse primers to the *B. multivorans recA* gene and to the human *GAPDH* gene as outlined in [7] were used and amplification was carried out in a final volume of 20 µL containing 10 µL SYBR Green PCR Mix (Roche Diagnostics Ltd, Burgess Hill, England), 379 nM *GAPDH* primers or 190 nM *RecA* primers, and 4 µL of DNA. The plate was centrifuged briefly for 1 min at 700 *g*. Real-time PCR was carried out on 7300 Real-Time PCR System (Applied Biosystems, Carlsbad, CA, USA). The samples were amplified in triplicate as follows: 10 min at 95 °C for denaturation, followed by 40 cycles at 95 °C for 15 s and 66 °C for 1 min. The data was collected during the 66 °C annealing/elongation phase. A dissociation step was carried out for melting curve analysis. The Pfaffl equation [13] was used to determine results incorporating the relative efficiencies of amplification of the target and reference genes. Real-time PCR efficiencies (E) were acquired using a five-point standard curve with both *GAPDH* and *recA* gene primers to determine the slope. The corresponding efficiencies were calculated according to the following equation: Efficiency (E) = (10^(−1/slope)^). Using these calculated efficiencies, the relative amount of bacterial DNA was determined compared to human *GAPDH* based on the cycle threshold values of bacterial versus human genes [13].

### 3.4. Sugar Competition Assays

Sterile solutions of bivalent lactosides (4.7 to 90 μM) or lactose were prepared in MEM. An overnight culture of LMG13010 was grown to an OD_600 nm_ of 0.6 and diluted to a final concentration of 5 × 10^6^ CFU·mL^−1^. Bacteria were pre-incubated with the sugar solutions for 30 min at 37 °C with gentle shaking and then applied to the 16HBE14o− cells. The plate was centrifuged at 700 *g* for 5 min and incubated at 37 °C in the presence of 5% CO_2_ for 30 min. Cells were washed vigorously three times with PBS to remove unbound bacteria. Real-time PCR was used for relative quantification and data for each sample in the presence of lactoside were compared with that of the 0.5% DMSO control.

## 4. Conclusions

The flexible bivalent lactoside showed promise in inhibition of attachment of *B. multivorans* to human lung cells. These encouraging results open the possibility to explore an anti-adhesion approach for *Burkholderia* infection. Little is known about the structural motifs that regulate adhesion in this pathogen, so, at present, it is difficult to carry out “rational design” for specific ligands. Hence we are continuing to investigate structurally diverse synthetic glycoconjugates, and the influence of the presentation of the carbohydrates in the different scaffolds in inhibiting/promoting adhesion may help to elucidate the structural basis of these processes.
